# Prevalence of dementia diagnosis in Sweden by geographical region and sociodemographic subgroups: a nationwide observational study

**DOI:** 10.1016/j.lanepe.2024.101029

**Published:** 2024-08-16

**Authors:** Mozhu Ding, Stina Ek, Emil Aho, Linus Jönsson, Katharina Schmidt-Mende, Karin Modig

**Affiliations:** a Unit of Epidemiology, Institute of Environmental Medicine, Karolinska Institutet, Stockholm, Sweden; b Division of Neurogeriatrics, Department of Neurobiology, Care Sciences and Society, Karolinska Institutet, Solna, Sweden; c Academic Primary Health Care Centre, Stockholm Region, Stockholm, Sweden; d Division of Family Medicine and Primary Care, Department of Neurobiology, Care Sciences and Society, Karolinska Institutet, Huddinge, Sweden

**Keywords:** Dementia diagnosis, Prevalence, Geographical variation, Primary care, Specialist care

## Abstract

**Background:**

Although dementia incidence has decreased in high-income countries, it is important to monitor the prevalence of dementia and identify potential underdiagnosis in population subgroups. This study provides the most up-to-date prevalence of dementia diagnosis in Sweden, by geographical regions and sociodemographic groups.

**Methods:**

We identified all individuals aged ≥62 years, registered and alive in Sweden at the end of 2022 (n = 2.48 million). Dementia diagnoses were identified using ICD-9/10 codes in the National Patient Register since 1987, as well as anti-dementia drug use from the Prescribed Drug Register since 2005.

**Findings:**

At the end of 2022, 3.7% (92,293/2,483,798) of people aged ≥62 years in Sweden had a dementia diagnosis from specialist care or drug prescriptions and varied from 0.6% in ages 62–69 to 14.8% in ages ≥90. The prevalence of cognitive impairment diagnosis was 2.5%. There was some geographical variation in the prevalence of dementia diagnosis, with a larger proportion of diagnoses coming from drug prescriptions than from specialist care in northern Sweden. While people born abroad and people without a close relative had a slightly higher prevalence of dementia diagnosis than Swedish born and those with close relatives, the prevalence was substantially lower for people living alone than for cohabiting individuals.

**Interpretation:**

Comparing case estimates from previous screening cohorts, our results suggest underdiagnosis of dementia in the general older population, particularly among people who live alone. In more rural areas with lower availability of memory clinics, primary care may play an important role in diagnosing older adults with dementia.

**Funding:**

Swedish Research Council for Health, Working Life and Welfare; 10.13039/501100004359Swedish Research Council; Region Stockholm.

**Research in context**

**Evidence before this study**

Receiving a dementia diagnosis is important for the patient to get access to adequate care and for policy makers to plan for and evaluate care utilization. While many studies have reported the prevalence of dementia in local research cohorts with in-person screening, less is known about the prevalence of clinically diagnosed dementia. We searched Web of Science for articles published until May 29, 2024, using the terms “dementia diagnosis”, “prevalence”, “specialist care OR primary care”, “geographical variations”, “sociodemographic” with no restrictions on language and dates. We identified five studies that reported nationwide prevalence of clinically diagnosed dementia, mostly in the United States and Germany, out of which only one investigated geographical variation in the prevalence of dementia diagnosis and no study examined sociodemographic differences.

**Added value of this study**

Sweden is known for its high-quality healthcare registers. To our knowledge, this is the first study to provide nationwide figures on the prevalence of dementia diagnosis based on diagnoses from specialist care and anti-dementia drug prescriptions, separately and together, in the total older population and across geographical regions. We were also able to describe dementia diagnosis in different sociodemographic subgroups, with the indication of potential underdiagnosis in some groups.

**Implications of all the available evidence**

Dementia is underdiagnosed in the older population, particularly among older adults who live alone. In more rural areas with lower availability of memory clinics, primary care may play an important role in diagnosing older adults with dementia.

## Introduction

Dementia is a public health concern, as it is one of the most common diseases in old age and a major cause of disability and mortality.^1^ It has a lasting and detrimental influence on patients, their families, and society at large. In times of population aging, it is important on a societal level to monitor dementia diagnosis prevalence in the population and to evaluate if diagnostics differ between population subgroups. For the individual patient, it is important to get a timely and accurate diagnosis to access adequate care.

Existing studies on dementia prevalence are typically based on in-person testing in research cohorts selected from urban areas, which are then combined with demographic data to estimate prevalence for larger populations.^2^^,^^3^ For instance, the Global Burden of Disease Study, which is based on community samples from different countries, estimated that 57 million people suffered from dementia worldwide in 2019 and the number for Sweden was 153,805.^2^ However, it is hard to know how many of these individuals received a dementia diagnosis in a clinical setting and were later treated. Therefore, complementary analyses of dementia prevalence based on real world data about dementia diagnosis is warranted. To the best of our knowledge, there are no nationwide studies in Sweden, and very few in the rest of the world,^4^^,^^5^ that explored the prevalence of dementia diagnosis in the total population, and how it differs in subgroups. Such studies could inform about actual estimates of dementia diagnosis and suggest vulnerable groups that are potentially under-diagnosed.

By utilizing information from etiological studies of risk factors for dementia, we can expect higher or lower dementia prevalence in different population subgroups. For example, previous studies have shown that low education and social isolation increase the risk for dementia,^1^ which would result in a higher prevalence for these groups. At the same time, being foreign born, having low education, and living alone have been associated with a lower likelihood of receiving a dementia diagnosis as well as drug treatment after dementia diagnosis,^6^, ^7^, ^8^ which would counteract the previous effect. Therefore, the distribution of dementia diagnosis prevalence can be influenced by both the underlying risk of dementia and factors influencing the likelihood of a diagnosis. How these factors manifest and translate into dementia diagnosis on the population level is so far not known. Moreover, in countries with universal healthcare such as Sweden, we can expect small variations in the diagnosis of dementia across geographical regions after taking out differences in population demographics. However, given that the 21 Swedish regions are self-governed, regional variations in care utilization and clinical practice of making a diagnosis may still result in regional differences in dementia prevalence,^9^, ^10^, ^11^ which is so far understudied.

Sweden is known for its high-quality healthcare registers, which is ideal for generating accurate estimations of dementia diagnosis. Utilizing nationwide data from specialized care and prescribed drugs, as well as regional data from primary care, this study aimed to 1) map the prevalence of dementia diagnosis at the end of 2022 in the entire Swedish older population and in the 21 Swedish regions, and 2) examine prevalence of dementia diagnosis in different sociodemographic subgroups. As cognitive impairment is a common stage before dementia onset, and with the hypothesis that some may remain at this diagnostic state, we also performed additional analyses examining the prevalence of cognitive impairment without a dementia diagnosis.

## Methods

### Study population

Data underlying this study was derived from the Aging and Health cohort, which was developed through linkage of several Swedish nationwide registers, covering all individuals in Sweden born before 1960 and followed until the end of 2022. Data from the Total Population Register was used to identify all individuals in the cohort who were alive at the end of 2022, in total 2,483,798 individuals aged 62 years and older. Through a unique personal identification number assigned to all individuals registered in Sweden, the Total Population Register was linked to individual-level data from national registers including the National Patient Register (since 1987), the Prescribed Drug Register (since 2005), the Longitudinal Integrated Database for Health Insurance and Labor Market Studies (LISA) (since 1990), the Multigeneration Register (since 1990), the Dwelling Register (since 2011), and the Swedish Social Service Register (since 2007). For Stockholm Region, primary care data is additionally available since 2013 via the Stockholm Region's Central Data Warehouse (VAL).

This research was register-based and did not involve direct contact with the study participants. It was performed in accordance with the ethical standards as laid down in the 1964 Declaration of Helsinki and its later amendments. Informed consent is not required for register-based studies, as the contribution of personal data to research is included in the contract between Swedish residents and the Swedish state provided that the research is ethically conducted.^12^ Ethical approval for this study was obtained from the Regional Ethics Committee in Stockholm (2011-136-31/5, 2021-02880).

### Diagnosis of dementia and cognitive impairment

We identified all diagnoses of dementia and cognitive impairment until December 31st, 2022 from the National Patient Register, which contains hospital discharge records from inpatient care at national level since 1987 and data on specialist outpatient care since 2001. Data on primary care is additional available for Stockholm Region, which includes all primary care visits from 2013 to 2022. Information retrieved from these registers includes the dates and discharge diagnoses of each visit which were coded according to the International Classification of Diseases (ICD) system. ICD-10 codes F00, F01, F02, F03, F05.1, G30 and ICD-9 codes 290, 294.1, 294.2, 331.0, 331.1 were used to identify dementia diagnosis. ICD-10 codes F06.7, R41, F10.7 and ICD-9 codes 780.9, 291.2 were used to identify diagnosis of cognitive impairment. In this study, individuals who received a cognitive impairment diagnosis but never a dementia diagnosis were considered to have cognitive impairment without dementia (hereafter referred to as cognitive impairment).

Beginning in July 2005, information on prescribed medications was available in the Prescribed Drug Register at national level, and all prescriptions were coded using the Anatomical Therapeutic Chemical (ATC) system. Use of any anti-dementia drugs (ATC code N06D) during 2005–2022 was retrieved from this register and individuals who picked up an anti-dementia drug at least once were considered as users and thus having dementia. Because primary care data was not available for entire Sweden in the current study, using anti-dementia drug prescriptions could serve as a proxy of additional dementia cases that were likely diagnosed in primary care but not captured by specialist care.

### Demographic and sociodemographic variables

Data on date of birth, country of birth, sex, highest attained education level, and family disposable income were retrieved from the LISA register. Education was categorized according to years of formal schooling into lower than high school (≤9 years), high school (10–12 years), and university (≥13 years). Family disposable income was dichotomized based on the age group-specific median. Information on living arrangements were retrieved from the Dwelling Register, which provides data on cohabitation status for people who live at home, as well as the Swedish Social Service Register, which provides data on care home services for older adults. All individuals were categorized as living alone at home, living with someone at home, and living in a care home. Data on family status were derived from the Multigeneration Register. Individuals cohabiting or having adult children were classified as having a close relative; individuals living alone or in a care home without adult children were classified as not having a close relative.

### Statistical analysis

First, the prevalence of dementia was calculated for the total Swedish population by taking all individuals alive on December 31st 2022 who ever received a dementia diagnosis in the National Patient Register or used anti-dementia drugs up until December 31, 2022 divided by the total population on December 31st 2022. The prevalence of dementia diagnosis was then calculated in the same way but separately for the 21 Swedish regions, by four sociodemographic indicators (i.e., place of birth, living arrangements, family status, and family disposable income), and by four age groups (62–69, 70–79, 80–89, and ≥90 years). To make the estimates comparable across subgroups of age, sex, and education, all prevalences were standardized to the age, sex, and education structure of the Swedish older population in 2022. In addition, logistic regressions adjusting for age, sex, and education were used to assess statistical differences in the prevalence of dementia diagnosis across socio-demographic subgroups, as well as by sex in each geographical region. The same analyses were performed for cognitive impairment. For dementia diagnosis prevalence, secondary analyses were performed accounting separately for specialist diagnosis from National Patient Register and use of anti-dementia drugs.

As a sensitivity analysis, dementia diagnoses from primary care were additionally added for the Stockholm Region where primary care data was available, to examine to what extent dementia diagnoses were found in specialist care, primary care, and drug use, respectively.

All statistical analyses were performed using Stata 16 (StataCorp LLC, College Station, TX 77845, USA) and RStudio version 2023.06.2.

### Role of the funding source

The funders had no role in the study design, data collection and analysis, interpretation of data, writing of the manuscript, and the decision to submit the manuscript for publication.

## Results

Among the 2,483,798 individuals aged ≥62 years and alive at the end of 2022 in Sweden, 92,293 received a diagnosis of dementia up until December 31st, 2022, equivalent to a prevalence of 3.7%. The prevalence of cognitive impairment diagnosis was 2.5%, with 62,374 individuals ever receiving a diagnosis of cognitive impairment but no diagnosis of dementia. Table 1 shows the number and crude prevalence of dementia diagnosis by age and sociodemographic groups. The prevalence of dementia diagnosis increased sharply with age, from 0.6% in 62–69 years to 14.8% in ≥90 years. The diagnosis prevalence decreased with increasing education level for all age groups, except for ≥90 years where the prevalence was the same for high education as for the low.

**Table 1:** Number of people with dementia (based on diagnosis in specialist care or prescription of anti-dementia drug) in Sweden by the end of 2022, stratified by age groups and population characteristics.

Characteristics	62–69 years		70–79 years		80–89 years		≥90 years	
	N (%)	Dementia (% out of N)	N (%)	Dementia (% out of N)	N (%)	Dementia (% out of N)	N (%)	Dementia (% out of N)
Overall	880,622	5432 (0.6)	1,020,981	27,853 (2.7)	480,145	43,887 (9.1)	102,050	15,121 (14.8)
Sex								
Male	438,169 (49.8)	2725 (0.6)	492,898 (48.3)	13,019 (2.6)	210,472 (43.8)	17,549 (8.3)	31,669 (31.0)	3901 (12.3)
Female	442,453 (50.2)	2707 (0.6)	528,083 (51.7)	14,834 (2.8)	269,673 (56.2)	26,338 (9.8)	70,381 (69.0)	11,220 (15.9)
Education level^a^								
Lower than high school	151,161 (17.2)	1259 (0.8)	248,754 (24.4)	7719 (3.1)	175,883 (36.6)	16,681 (9.5)	49,401 (48.4)	7229 (14.6)
High school	412,522 (46.8)	2510 (0.6)	442,390 (43.3)	12,334 (2.8)	183,349 (38.2)	16,908 (9.2)	32,669 (32.0)	4994 (15.3)
University or above	307,239 (34.9)	1567 (0.5)	318,057 (31.2)	7464 (2.4)	112,842 (23.5)	9607 (8.5)	17,730 (17.4)	2596 (14.6)
Place of birth								
Born in Sweden	716,604 (81.4)	4268 (0.6)	886,224 (86.8)	23,908 (2.7)	418,696 (87.2)	37,755 (9.0)	90,293 (88.5)	13,206 (14.6)
Born outside of Sweden	164,018 (18.6)	1164 (0.7)	134,757 (13.2)	3945 (2.9)	61,449 (12.8)	6132 (10.0)	11,757 (11.5)	1915 (16.3)
Living arrangement								
Live alone at home	251,069 (28.5)	1455 (0.6)	327,515 (32.1)	6216 (1.9)	207,983 (43.3)	10,023 (4.8)	53,052 (52.0)	2954 (5.6)
Live with someone at home	623,779 (70.8)	2561 (0.4)	674,014 (66.0)	12,399 (1.8)	234,904 (48.9)	13,648 (5.8)	21,171 (20.8)	1744 (8.2)
Live in a care home	5774 (0.7)	1416 (24.5)	19,452 (1.9)	9238 (47.5)	37,258 (7.8)	20,216 (54.3)	27,827 (27.2)	10,423 (37.5)
Family status^a^								
Have a close relative	667,414 (75.8)	3264 (0.5)	737,382 (72.2)	17,619 (2.4)	292,907 (61.0)	23,975 (8.2)	36,199 (35.5)	4743 (13.1)
Do not have a close relative	178,585 (20.3)	1841 (1.0)	277,058 (27.1)	10,048 (3.6)	186,689 (38.9)	19,828 (10.6)	65,752 (64.4)	10,362 (15.8)
Family disposable income								
Below median	439,262 (50.0)	3893 (0.9)	509,694 (50.0)	16,985 (3.3)	239,764 (50.0)	23,330 (9.7)	50,920 (50.0)	7574 (14.9)
Above median	439,398 (50.0)	1538 (0.4)	510,153 (50.0)	10,851 (2.1)	240,004 (50.0)	20,533 (8.6)	51,079 (50.0)	7545 (14.8)

a Missing data in education level and family status accounts for 1.3% and 1.7%, respectively.

**Fig. 1:**
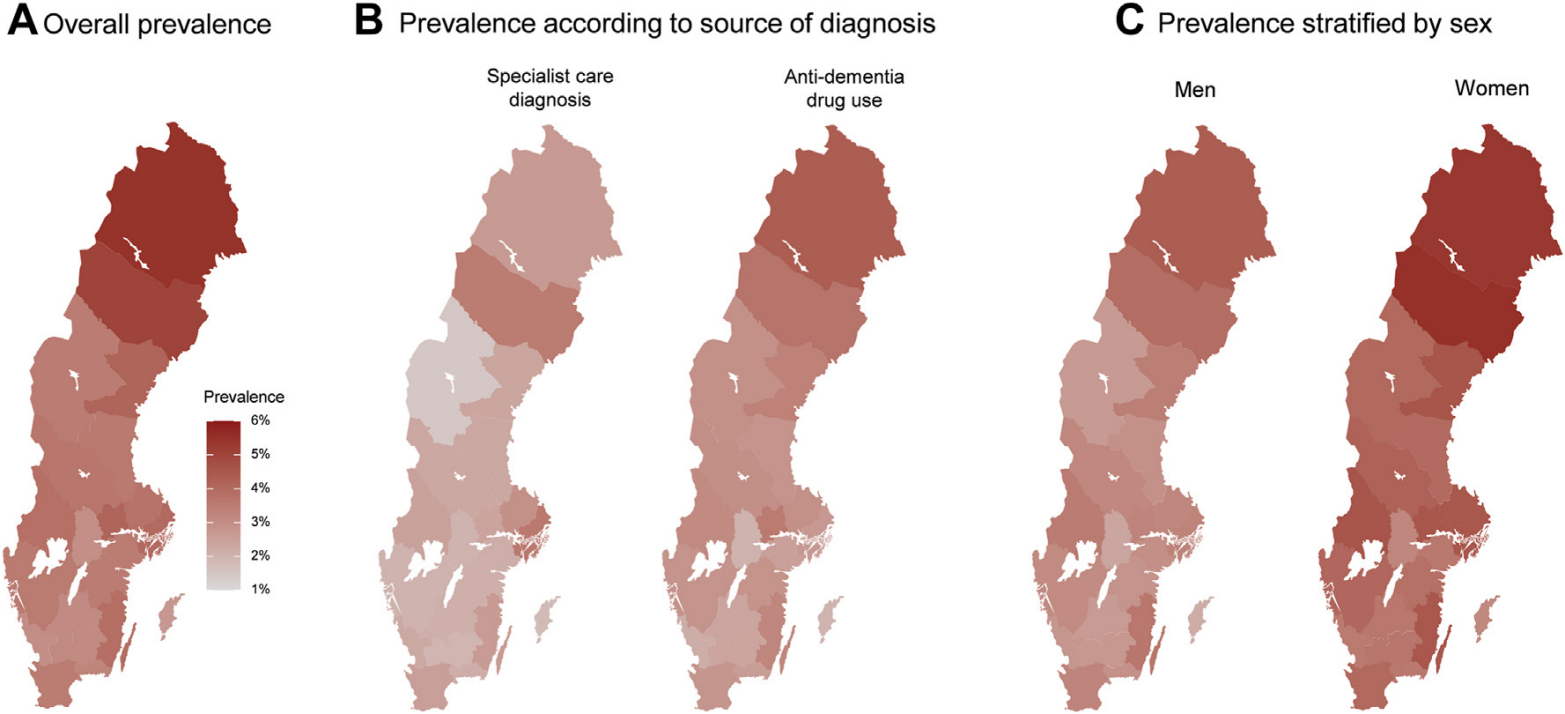
Standardized prevalence of dementia (aged ≥62 years) in 21 Swedish regions at the end of 2022. A) Overall prevalence of dementia based on diagnoses from specialist care and/or use of anti-dementia drugs; B) prevalence of dementia according to source of diagnosis, from either specialist care (left) or use of anti-dementia drugs (right); C) prevalence of dementia stratified by sex. All regional prevalences are standardized to the age, sex, and education structure of the total Swedish population in 2022.

Fig. 1 shows the standardized prevalence of dementia diagnosis across the 21 Swedish regions. Overall, there were some geographical differences in the prevalence of dementia diagnosis in Sweden (ranging between 2.7% and 5.5%) (Fig. 1A). The prevalence of dementia diagnosis in the two regions far north was slightly higher than others, i.e., 5.5% for Norrbotten Region and 5.1% for Västerbotten Region. When dementia was identified solely through visits to specialist care, the northern counties had similar prevalence as the rest, but when dementia drugs were considered, they had a higher prevalence instead, as shown in Fig. 1B in which the prevalence was separately calculated based on specialist diagnosis only and drug use only. Gotland island had the lowest diagnosis prevalence at 2.7%. The diagnosis prevalence in the other regions varied between 3.0% and 4.2%. The prevalence of dementia diagnosis was statistically significantly higher in women than in men in all regions except for Kalmar, Blekinge, Skåne, and Halland Region (Fig. 1C, Supplementary Table S1). The prevalence of cognitive impairment diagnosis ranged from 1.4% to 3.5% across Sweden and was very similar among men and women (Supplementary Figure S1).

**Fig. 2:**
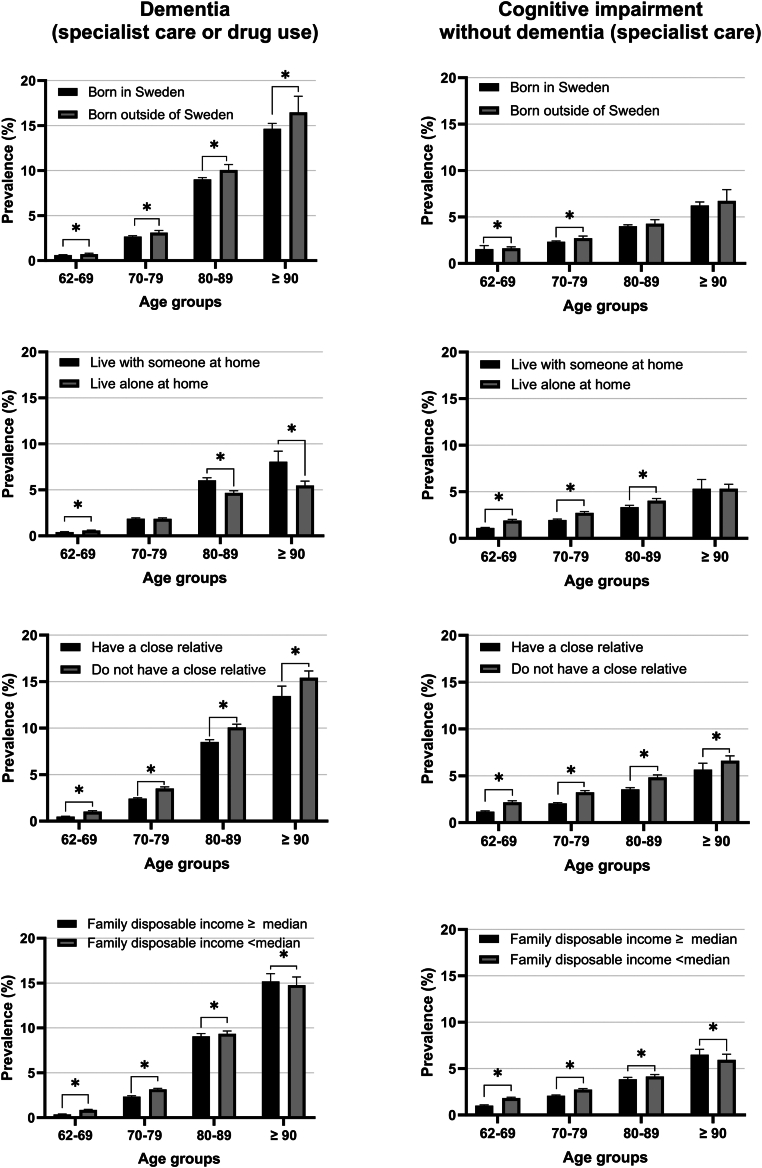
Standardized prevalence of dementia and cognitive impairment diagnosis in Sweden in 2022 stratified by age groups, place of birth, living arrangement, family status, and family disposable income. All prevalences are standardized to the age, sex, and education structure of the total Swedish population in 2022. ∗p < 0.05.

Fig. 2 shows the standardized prevalence of dementia diagnosis and cognitive impairment diagnosis by age and sociodemographic subgroup. Individuals that were born outside of Sweden had slightly higher prevalence of dementia diagnosis in all age groups than those born in Sweden (p < 0.05 for all age groups, Supplementary Table S2). For instance, for age group ≥90 years, the prevalence of dementia diagnosis was 16.5% in foreign-born individuals and 14.7% in Swedish born. The prevalence of dementia diagnosis was also slightly higher among people who did not have a close relative compared to those with a close relative (p < 0.05 for all age groups, Supplementary Table S2). With regard to living arrangements, people who were living at home had lower prevalence of dementia diagnosis compared to those who were living with someone at home, especially in age group 80–89 and ≥90 years (p < 0.05, Supplementary Table S2). The prevalence was similar between people with low or high family disposable income, albeit statistically significantly different. The prevalence of cognitive impairment diagnosis on the other hand did not differ substantially by sociodemographic subgroups, albeit statistically significant between certain subgroups (Supplemental Table S3). Sex-specific analyses on dementia diagnosis prevalence by sociodemographic groups yielded similar patterns as the main analyses (Supplementary Figure S2).

Limiting the population to Stockholm Region, where primary care diagnoses were additionally available, resulted in similar patterns by sociodemographic groups as in the nationwide analyses (Supplementary Figure S3). The overall prevalence of dementia diagnosis was 3.8% in Stockholm Region, which slightly increased to 4.1% after adding primary care diagnosis on top of specialist care diagnosis and drug use; the largest increase was observed in the oldest old (≥90 years), from 16.7% to 19.3% (Supplementary Figure S4). How dementia diagnoses were distributed among different settings in Stockholm Region is presented in Venn diagrams (Supplementary Figure S5). A total of 18,162 individuals had an existing dementia diagnosis in Stockholm Region at the end of 2022, and half (50.2%) of them were captured by all three sources – specialist care, primary care, and drug prescriptions. Only 4.9% of the diagnoses were captured only by primary care, which means that 95.1% of all dementia diagnoses were captured by specialist care and drug use. When stratified by age groups, however, the percentage of diagnoses captured by primary care only was 10.9% for people aged ≥85 years and 6.4% for people aged <85 years.

## Discussion

Utilizing data from the entire Swedish older population, we have the following key findings: 1) almost 4% of all individuals aged ≥62 years in Sweden were living with a dementia diagnosis at the end of 2022, and when accounting for age, sex, and education, there were some variations in the dementia diagnosis prevalence across geographical regions, 2) differences in the prevalence of dementia diagnosis across sociodemographic groups were small, however, the prevalence was substantially lower among live-alone individuals, and 3) there was a difference between the regions in the prevalence of dementia diagnosis identified from specialist care versus prescribed drugs. This indicates that in some regions there is likely a higher share of dementia patients diagnosed in primary care only.

To our knowledge, this is the first study to report the prevalence of dementia diagnosis in Sweden as a whole and across geographical regions. The true prevalence of dementia is always higher than can be identified from diagnoses. This is reflected by the fact that our estimates are lower than those from local clinical cohorts in which dementia was screened for and diagnosed with, for instance, the Standard Classification of Mental Disorders (DSM) criteria.^13^, ^14^, ^15^ For example, a cohort study of 3363 individuals reported that the prevalence of dementia in a central area of Stockholm was 7.1% for people aged ≥66 years and 29.8% for those aged ≥90 years, according to DSM-III-R criteria.^15^ We, on the other hand, reported 3.8% and 16.7% respectively according to diagnosis attained from specialist care and drug use. Nevertheless, reporting prevalence of diagnosed dementia is important as it reflects potential underdiagnosis. Studies have shown that the validity of dementia diagnosis in the Swedish National Patient Register, measured through positive predictive values, is high against the DSM-III-R criteria.^16^ This means that the vast majority of those who have a dementia diagnosis in the register indeed have dementia and substantial overestimation is unlikely. Yet, the registers capture only about 50% of individuals who receive a diagnosis in clinical cohort studies. It is therefore likely that nearly twice as many people in Sweden as reported in our study would have received a dementia diagnosis if they underwent diagnostic assessments. Indeed, the Global Burden of Disease Study estimated the total number of “true” dementia cases as 153,805 for Sweden in 2019,^2^ and the number of dementia diagnoses reported in our study is 92,293 for 2022. This indicates that approximately 40% of individuals with dementia in the general population are potentially undiagnosed, although the exact number of dementia cases for 2022 is unknown. However, because the underlying risk of dementia may have declined in high-income counties, as indicated by a decrease in the incidence of dementia cases in previous cohort studies,^17^^,^^18^ it is possible that the number of true dementia cases in the general population nowadays are lower than projected by previous studies using older data. With regards to cognitive impairment, there is a lack of evidence about the validity of specialist and primary care diagnosis of cognitive impairment in Sweden. However, given that previous cohort studies with in-person screening reported the prevalence to be approximately 20% among people aged ≥70 years,^19^^,^^20^ our results indicate even greater underdiagnosis of cognitive impairment in specialist and primary care.

When accounting for differences in age, sex, and education, there were some but not substantial variations in the prevalence of dementia diagnosis across the Swedish regions on an absolute scale. This is reassuring but perhaps not surprising given that the Swedish healthcare system is homogeneous with low levels of private insurance, few providers outside the public reimbursement system, and low patient cost sharing. However, the two regions far north showed a higher diagnosis prevalence than the other regions. This is in line with a study using the Swedish Twin Register which found a higher prevalence of dementia in the north, but this was not explained by regional differences in genetic or shared lifestyle factors such as diet.^10^ A north-south difference has also been found in Finland.^21^ Previous data has suggested that early-life rural living is a risk factor for dementia,^9^^,^^22^ however, the diagnosis prevalence patterns from our and other studies did not fit entirely with urban and rural areas. Moreover, as we have adjusted for age, sex, and education differences, the sensitivity of National Patient Register should be similar across the regions. Other explanations could be regional differences in diagnostic procedure, awareness of dementia in the community, medical practice norms that influence the desirability of a dementia diagnosis, and care utilization.^23^ Indeed, our data shows higher rates of anti-dementia drug prescriptions in the northern regions but not diagnosis from specialist care. This suggests that drug prescriptions might have been initiated in primary care without referral to specialist care to a higher extent in the north.

Previous etiological studies have consistently reported higher risk of developing dementia among individuals with lower socioeconomic positions.^24^^,^^25^ Individual-level deprivation (e.g., low household income and wealth) and lower education was associated with 20–90% higher risk of dementia in high-income countries.^24^^,^^26^^,^^27^ Similarly, social isolation, including living alone and having no one in life to discuss important matters, was found to be associated with ∼50% increased dementia risk in previous studies.^28^^,^^29^ Given the magnitude of these associations, it may stand to reason that the prevalence of dementia diagnosis would also differ substantially by these factors. However, in this study, the prevalence of dementia diagnosis did not differ substantially by family income and was only slightly higher among people who were foreign-born or without a close relative compared to their counterparts; the absolute difference was even smaller for the prevalence of cognitive impairment diagnosis. It is therefore likely that factors related to healthcare seeking behaviors and the likelihood of a diagnosis may have had a greater influence on the dementia diagnosis than etiological factors affecting the underlying risk of dementia. People with higher socio-economic position (e.g., higher family income and education) may have better understanding about dementia and are more aware of early signs of cognitive decline, leading them to consult a physician earlier. Systemic reviews have found that education deficits were a major factor in dementia diagnosis delay,^30^ and a recent study from Danmark reported that those with higher household income often received earlier dementia diagnosis and, if diagnosed, had less severe dementia stage.^26^ Moreover, although population surveys reported a higher risk of developing dementia among all migrant groups in Europe,^31^ register-based studies often show opposing results including a Swedish register-based study reporting a lower incidence of dementia diagnosis among foreign-born individuals until 2012^32^ and a similar Norwegian study showing a lower prevalence in 2008.^33^ It is therefore plausible that even though dementia risk is elevated in these vulnerable groups, underdiagnosis of dementia has led to a diagnosis prevalence lower than expected. Living alone and not having a close relative were also associated with a lower likelihood of receiving dementia diagnostic investigations.^7^ We observed a much lower prevalence of dementia diagnosis among people living alone compared to those living with someone at home, particularly in the two older age groups. While dementia can be underdiagnosed in live-alone individuals, it is also likely that older adults who lived alone but with later-stage dementia had already moved to care homes, as reflected by a high prevalence of dementia diagnosis among care home residents in our study. Indeed, having a dementia diagnosis is one of the most important and common criteria of moving into a care home, however, studies have suggested potential underdiagnosis among care home residents who develop dementia.^34^

Primary care plays a unique and essential role in diagnosing and treating people with dementia, as it is often the first point of contact with healthcare. Yet, as central as primary care is to early detection of dementia, underdiagnosis of dementia has been reported to be common in primary care.^30^ Barriers to dementia diagnosis in primary care have been described in previous studies, including declining cognitive assessments from patients' side and lack of time, tools, and training from providers’ side.^35^ In Stockholm, we found that 10.9% of older people aged ≥85 years with dementia are diagnosed in primary care only, without contact with specialist care. This proportion increases to 14.4% if drug prescriptions in primary care are considered. As the primary care data was only available from 2013 and onwards, diagnoses before 2013 were not captured, which could further underestimate this proportion. The share of dementia diagnosed in primary care might be even higher in the northern part of Sweden given their higher share of anti-dementia drug prescriptions. Nevertheless, our results mark the important role of primary care in making a dementia diagnosis, especially for older individuals.

Strengths of this study include the national coverage of specialist care register and regional coverage of primary care register, enabling a thorough and up-to-date investigation of dementia and cognitive impairment diagnosis in Sweden. To the best of our knowledge, we are also the first to explore dementia diagnosis in population subgroups and different care settings. However, several limitations should be considered. First, as this is a cross-sectional study with a descriptive methodology, we did not attempt to sort out the order of events between sociodemographic factors and dementia diagnosis. Therefore, we cannot make inference on whether the presence or absence of these factors are associated with dementia risk. The focus of this study is rather to imply potential underdiagnosis in the total population and socio-demographic subgroups for which healthcare seeking behaviors differ the most, leading to differential likelihood of receiving a dementia diagnosis. We therefore did not consider other risk factors such as cardiovascular diseases and lifestyle factors (e.g., alcohol consumption) that are less likely to be related to the likelihood of a diagnosis. Moreover, given the nature of register-based study, to know the exact extent of dementia underdiagnosis in different population subgroups and geographical regions is not possible. Although most individuals with dementia will eventually receive a dementia diagnosis, they may be diagnosed at different severity stages and underdiagnosis may more likely concern people with early-stage dementia. Third, as current anti-dementia drugs are mostly used to treat Alzheimer's disease, and no specific drugs are yet available for vascular dementia, we could have missed to identify certain vascular dementia diagnoses by drug prescriptions alone. Finally, as we lack primary care data for all regions except for Stockholm, the true number and prevalence of dementia diagnosis may be higher than reported here. Although only 5% of all dementia diagnoses in Stockholm were captured by primary care alone, it is possible that a higher share of dementia cases is diagnosed in primary care alone for other regions due to a lower availability of memory clinics.

In Sweden, at least 92,300 older individuals aged ≥62 years were living with a dementia diagnosis at the end of 2022, which translates into a prevalence of 3.7%. Comparing case estimates from screening cohorts, our results indicate that dementia is underdiagnosed in the general population. The lower dementia diagnosis prevalence among individuals living alone suggests certain underdiagnosis in this group since living alone has been reported to be a risk factor for developing dementia. The similar diagnosis prevalence across other socioeconomic groups might indicate the same. The higher share of dementia identified from drug prescriptions rather than diagnosis in specialist care in the northern part of Sweden indicates that part of the diagnoses is only set in primary care.

## Contributors

MD and KM conceptualized and designed the study. MD conducted the data analyses and drafted the manuscript. All authors contributed to the interpretation of results and critically reviewed the manuscript. MD and KM have directly accessed and verified the underlying data reported in the manuscript.

## Data sharing statement

Due to the General Data Protection Regulation in Sweden, the pseudo-anonymized personal data underlying this study cannot be shared publicly. Access to the data and the codes for data analyses can be permitted to external researchers after ethical vetting and establishment of a collaboration agreement. Contact the corresponding author for questions about data sharing (MD).

## Editor note

The Lancet Group takes a neutral position with respect to territorial claims in published maps and institutional affiliations.

## Declaration of interests

We declare no competing interests.
