# Supplementary material for: Prevalence of dementia diagnosis in Sweden by geographical region and sociodemographic subgroups: a nationwide observational study
Source: Lancet Reg Health Eur. 2024 Aug 16;45:101029. doi: 10.1016/j.lanepe.2024.101029 (PMC11378931; doi:10.1016/j.lanepe.2024.101029)
Supplement: Supplementary materials [file mmc1.docx]

**
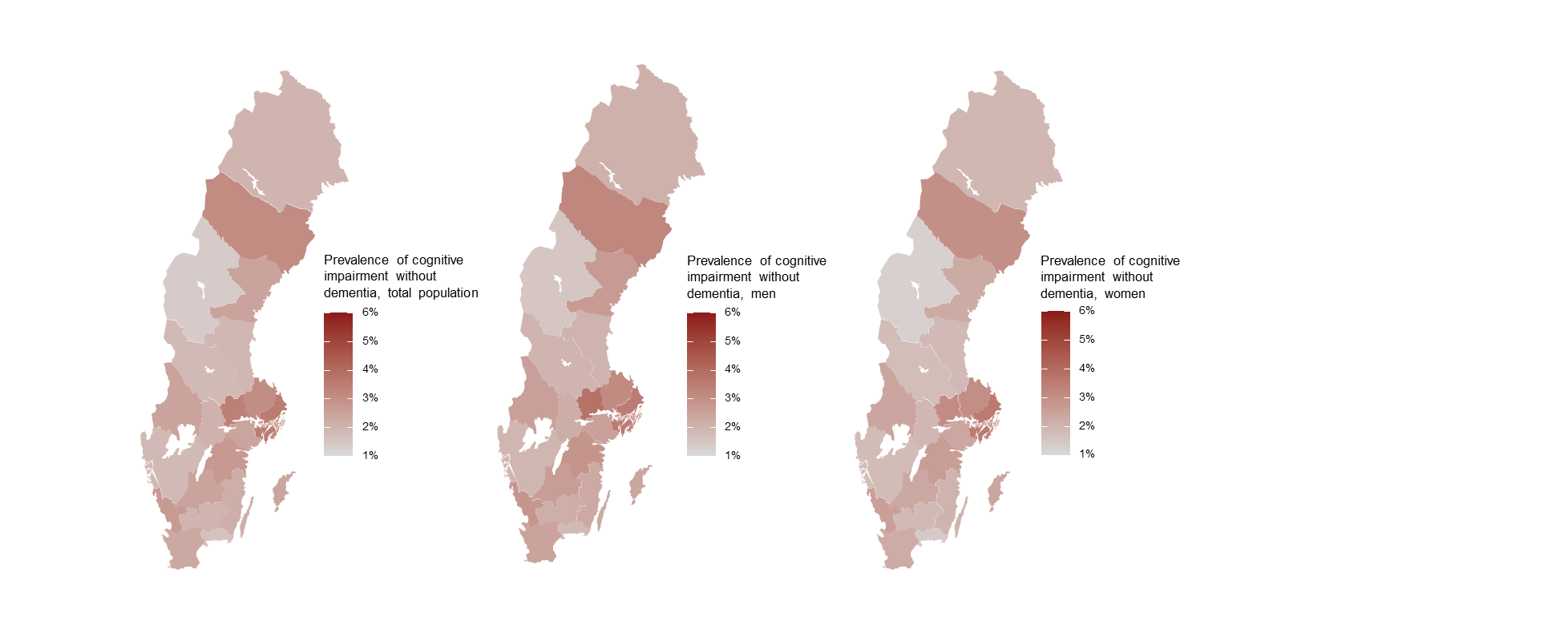
**

**Supplementary Figure 1**. Standardized prevalence of cognitive impairment based on specialist care diagnosis among 21 Swedish regions in 2022, in the total Swedish population (left) and stratified by men (middle) and women (right). All prevalences are standardized to the age, sex, and education structure of the total Swedish population in 2022.

**
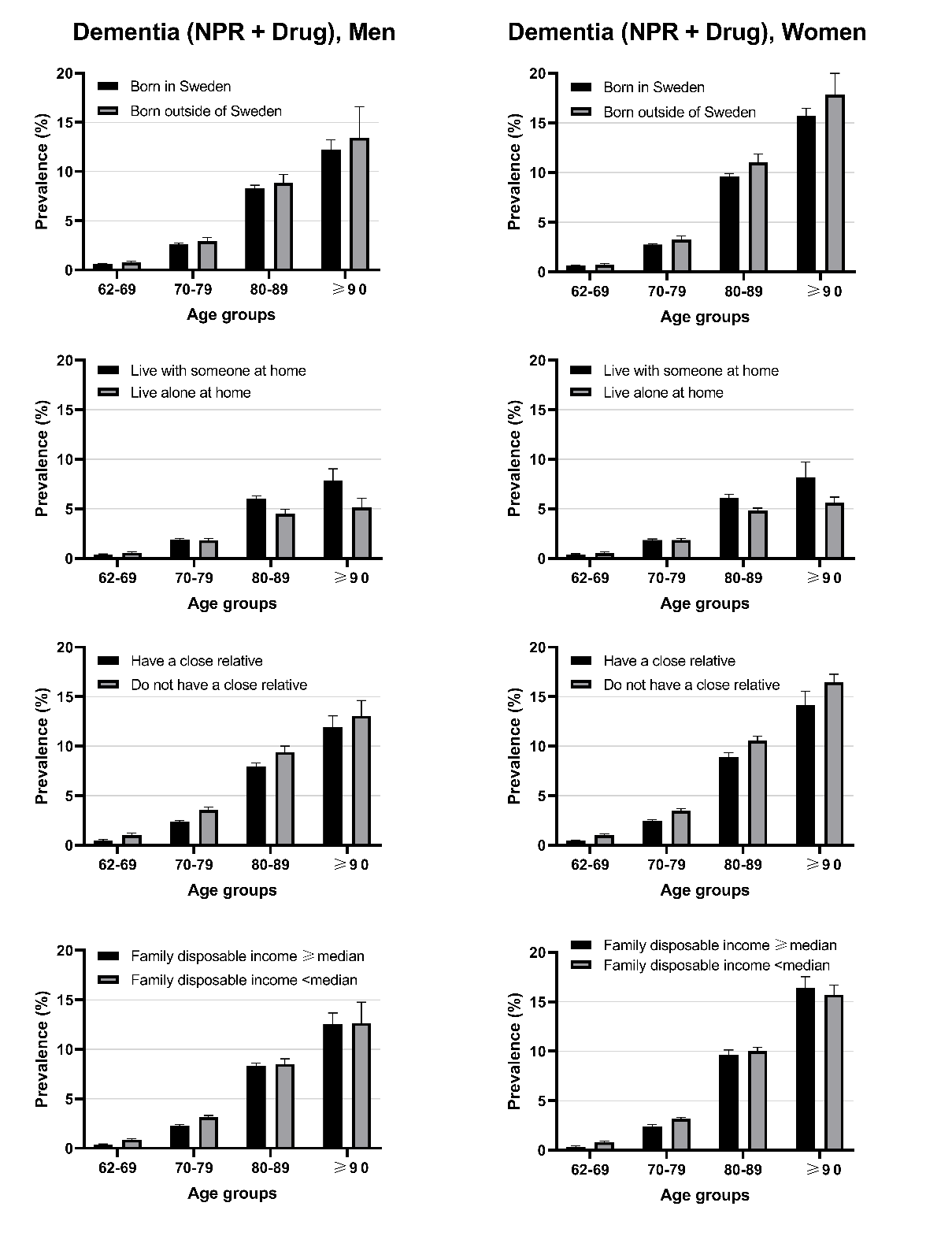
**

**Supplementary Figure 2.** Standardized prevalence of dementia diagnosis in Sweden by the end of 2022 in men (left) and women (right), stratified by age groups, place of birth, living arrangement, family status, and family disposable income. All prevalences are standardized to the age and education structure of the total Swedish population in 2022. NPR=National Patient Register, Drug= Prescribed Drug Register.


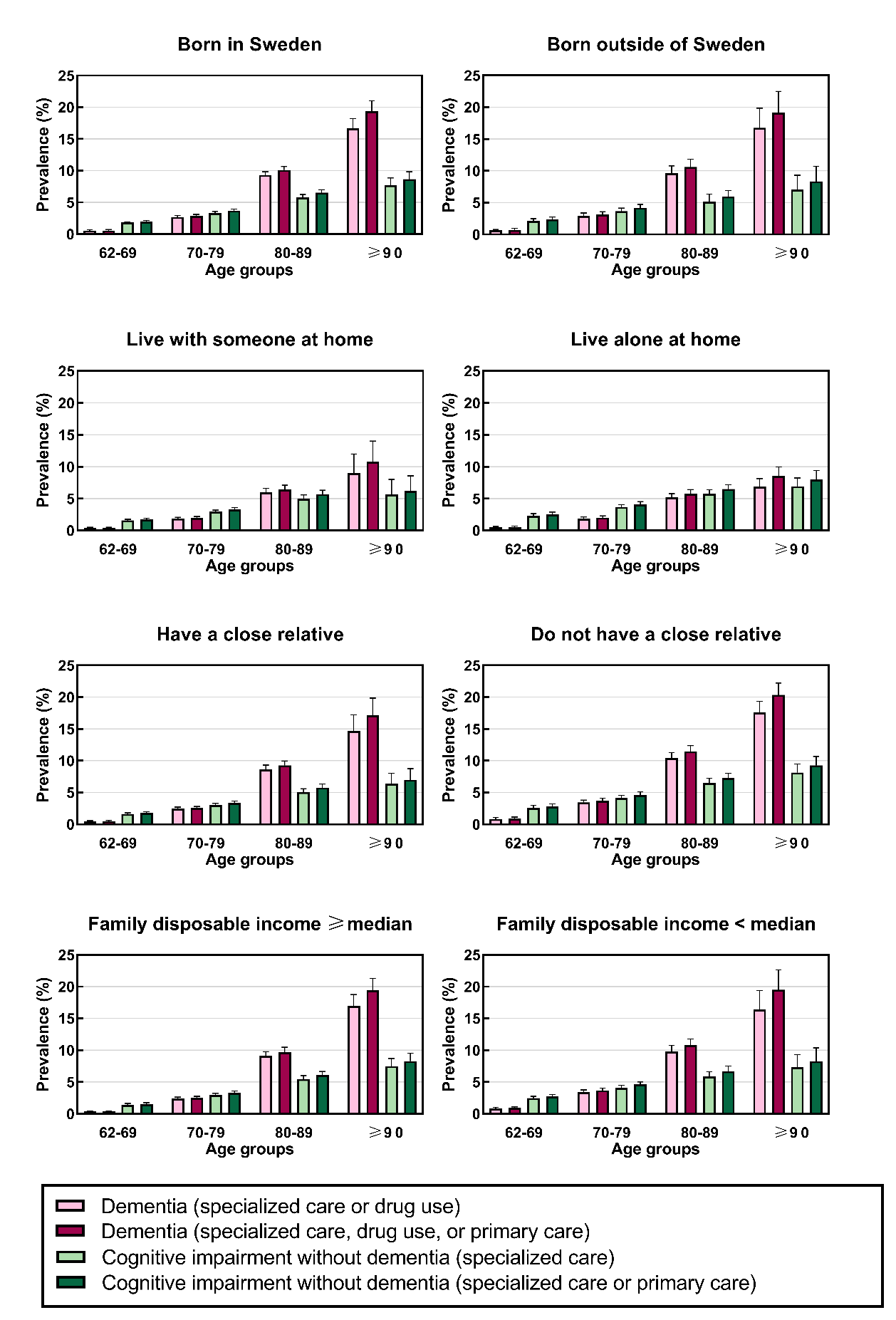


**Supplementary Figure 3**. Standardized prevalence of dementia and cognitive impairment diagnosis in Stockholm in 2022 stratified by age groups, place of birth, living arrangement, family status, and family disposable income. All prevalences are standardized to the age, sex, and education structure of the total Swedish population in 2022.

**
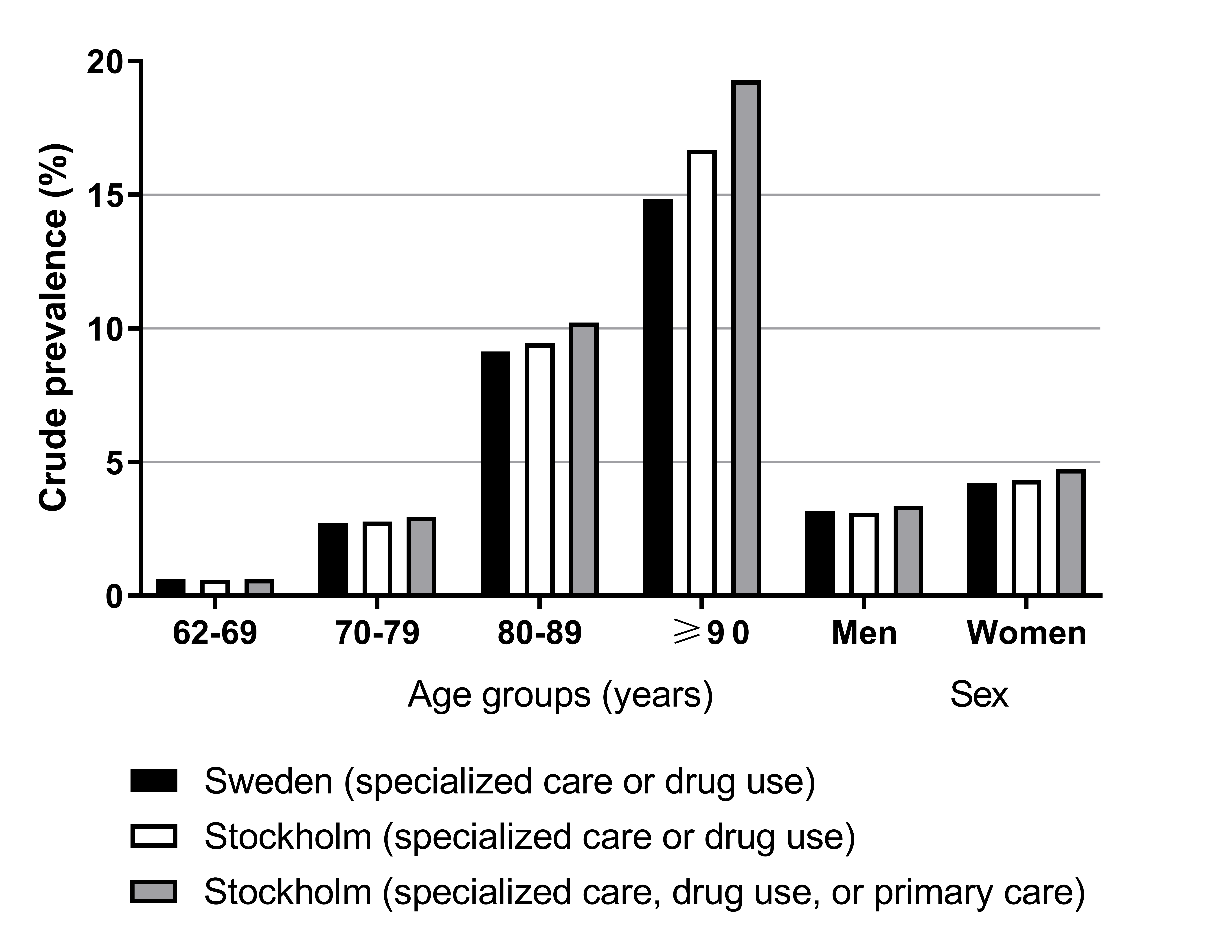
**

**Supplementary Figure 4**. Crude prevalence of dementia diagnoses in Sweden and Stockholm Region based on different registers, stratified by age and sex.


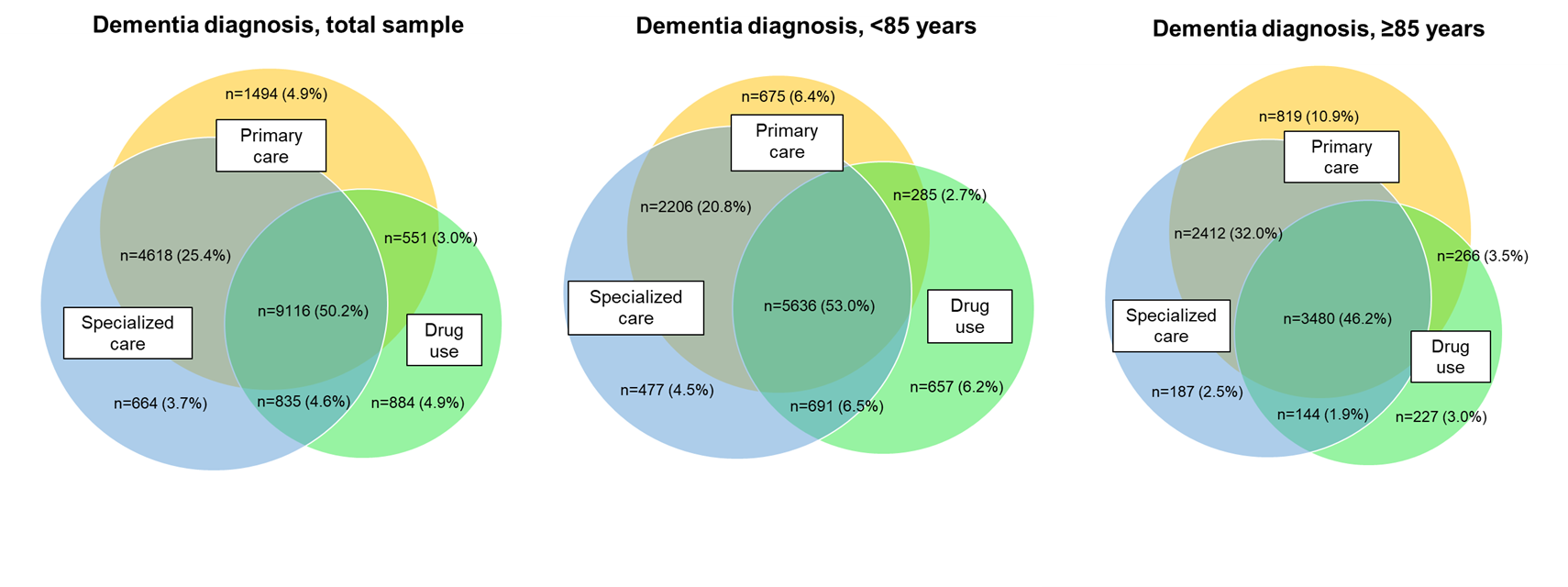


**Supplementary Figure 5**. Number and proportion of dementia diagnoses identified from specialized care (blue bubble), use of anti-dementia drugs (green bubble), or primary care (yellow bubble), respectively, in the total sample and by two age groups.

**Supplementary Table 1**. Standardized prevalence and odds ratios of dementia diagnosis by geographical regions and sex.

| Swedish geographical regions | Men | | Women | | OR (95% CI) of dementia diagnosis, women vs. men |
| --- | --- | --- | --- | --- | --- |
|  | N | Standardized prevalence (%)^*^ | N | Standardized prevalence (%)^*^ |  |
| Stockholm | 218,024 | 3.28 | 252,924 | 4.52 | 1.12 (1.08-1.16)^a^ |
| Uppsala | 41,050 | 3.21 | 45,901 | 4.49 | 1.13 (1.05-1.21)^a^ |
| Södermanland | 37,938 | 3.01 | 41,958 | 3.61 | 1.09 (1.01-1.18)^b^ |
| Östergötland | 53,871 | 2.86 | 59,967 | 3.84 | 1.12 (1.05-1.20)^a^ |
| Jönköping | 42,792 | 2.64 | 47,054 | 3.66 | 1.15 (1.07-1.24)^a^ |
| Kronoberg | 24,227 | 2.76 | 26,062 | 3.69 | 1.13 (1.02-1.25)^b^ |
| Kalmar | 34,696 | 3.65 | 38,000 | 4.50 | 0.99 (0.92-1.07) |
| Gotland | 8,919 | 2.17 | 9,870 | 3.14 | 1.20 (1.00-1.44)^b^ |
| Blekinge | 20,974 | 2.92 | 23,106 | 3.60 | 1.02 (0.92-1.13) |
| Skåne | 151,741 | 3.26 | 171,269 | 4.04 | 1.00 (0.96-1.04) |
| Halland | 41,987 | 2.79 | 46,556 | 3.54 | 1.02 (0.94-1.10) |
| Västra Götaland | 191,755 | 3.08 | 214,024 | 4.09 | 1.08 (1.04-1.12)^a^ |
| Värmland | 37,869 | 3.49 | 42,270 | 4.58 | 1.10 (1.02-1.19)^b^ |
| Örebro | 36,237 | 2.39 | 40,634 | 3.22 | 1.12 (1.02-1.22)^b^ |
| Västmanland | 33,432 | 3.27 | 37,206 | 4.55 | 1.09 (1.01-1.18)^b^ |
| Dalarna | 40,525 | 3.19 | 43,402 | 4.22 | 1.11 (1.03-1.19)^b^ |
| Gävleborg | 38,884 | 2.94 | 42,458 | 3.98 | 1.18 (1.09-1.27)^a^ |
| Västernorrland | 33,163 | 3.42 | 36,149 | 4.59 | 1.17 (1.08-1.26)^a^ |
| Jämtland | 17,691 | 2.68 | 18,774 | 4.03 | 1.27 (1.13-1.43)^a^ |
| Västerbotten | 32,996 | 3.91 | 35,915 | 5.51 | 1.24 (1.15-1.34)^a^ |
| Norrbotten | 34,437 | 4.35 | 37,091 | 5.34 | 1.09 (1.02-1.17)^b^ |

OR=odds ratio; CI=confidence interval. All odds ratios were adjusted for age and education. ^*^Standardized to the age and education structure of the Swedish older population in 2022. ^a^p<0.001; ^b^p<0.05.

**Supplementary Table 2**. Odds ratios (95% confidence interval) of dementia diagnosis according to place of birth, living arrangement, family status, and family disposable income, stratified by age groups.

| Sociodemographic characteristics | 62-69 years | 70-79 years | 80-89 years | ≥90 years |
| --- | --- | --- | --- | --- |
| Place of birth |  |  |  |  |
| Born in Sweden | Ref (1.00) | Ref (1.00) | Ref (1.00) | Ref (1.00) |
| Born outside of Sweden | 1.23 (1.15-1.21)^a^ | 1.17 (1.13-1.21)^a^ | 1.12 (1.09-1.18)^a^ | 1.12 (1.06-1.18)^a^ |
| Living arrangement |  |  |  |  |
| Live with someone at home | Ref (1.00) | Ref (1.00) | Ref (1.00) | Ref (1.00) |
| Live alone at home | 1.39 (1.29-1.47)^a^ | 0.99 (0.96-1.02) | 0.76 (0.74-0.78)^a^ | 0.66 (0.61-0.70)^a^ |
| Family status |  |  |  |  |
| Have a close relative | Ref (1.00) | Ref (1.00) | Ref (1.00) | Ref (1.00) |
| Do not have a close relative | 2.03 (1.92-2.15)^a^ | 1.46 (1.43-1.50)^a^ | 1.21 (1.18-1.23)^a^ | 1.17 (1.12-1.22)^a^ |
| Family disposable income |  |  |  |  |
| Below median | Ref (1.00) | Ref (1.00) | Ref (1.00) | Ref (1.00) |
| Above median | 0.42 (0.39-0.44)^a^ | 0.72 (0.71-0.74)^a^ | 0.96 (0.95-0.99)^b^ | 1.05 (1.01-1.09)^b^ |

All odds ratios were adjusted for age, sex, and education. ^a^p<0.001; ^b^p<0.01.

**Supplementary Table 3**. Odds ratios (95% confidence interval) of cognitive impairment diagnosis according to place of birth, living arrangement, family status, and family disposable income, stratified by age groups.

| Sociodemographic characteristics | 62-69 years | 70-79 years | 80-89 years | ≥90 years |
| --- | --- | --- | --- | --- |
| Place of birth |  |  |  |  |
| Born in Sweden | Ref (1.00) | Ref (1.00) | Ref (1.00) | Ref (1.00) |
| Born outside of Sweden | 1.16 (1.11-1.22)^a^ | 1.13 (1.09-1.17)^a^ | 1.03 (0.98-1.08) | 0.99 (0.91-1.07) |
| Living arrangement |  |  |  |  |
| Live with someone at home | Ref (1.00) | Ref (1.00) | Ref (1.00) | Ref (1.00) |
| Live alone at home | 1.71 (1.64-1.77)^a^ | 1.39 (1.35-1.43)^a^ | 1.22 (1.18-1.26)^a^ | 1.03 (0.65-1.12) |
| Family status |  |  |  |  |
| Have a close relative | Ref (1.00) | Ref (1.00) | Ref (1.00) | Ref (1.00) |
| Do not have a close relative | 1.89 (1.79-1.94)^a^ | 1.58 (1.54-1.41)^a^ | 1.36 (1.32-1.41)^a^ | 1.20 (1.13-1.27)^a^ |
| Family disposable income |  |  |  |  |
| Below median | Ref (1.00) | Ref (1.00) | Ref (1.00) | Ref (1.00) |
| Above median | 0.55 (0.53-0.58)^a^ | 0.76 (0.74-0.78)^a^ | 0.94 (0.91-0.97)^a^ | 1.12 (1.06-1.18)^a^ |

All odds ratios were adjusted for age, sex, and education. ^a^p<0.001.
